# Winners and losers: Emotional shifts across elections are conveyed by a politician’s smile

**DOI:** 10.1371/journal.pone.0301113

**Published:** 2024-04-29

**Authors:** Carl Senior, Patrick A. Stewart, Erik Bucy, Nick Lee

**Affiliations:** 1 School of Psychology Aston University, Birmingham, United Kingdom; 2 Department of Political Science, University of Arkansas, Fayetteville, Arkansas, United States of America; 3 College of Media & Communication, Texas Tech University, Lubbock, Texas, United States of America; 4 Warwick Business School, The University of Warwick, Coventry, United Kingdom; Yeditepe University, TURKEY

## Abstract

The human smile can convey both rewarding and affiliative social intent and thus has significant utility in politics, where the ability to bond with and reassure voters is vital to electoral success. We examine experimental evidence from the 2019 UK general election to investigate the influence of a politician’s reward or affiliative smile on voter emotions. It was hypothesised that the winner’s affiliative smile would engender positive affect across all partisan groups compared to the winner’s reward smile display. Participants from a nationally representative sample were shown campaign footage containing both types of smiles from the leaders of the main competing political parties both before and after the election. Increases in happiness and affinity were revealed across all partisan groups when shown footage of the eventual winner’s affiliative smile; at the same time, supporters of losing parties indicated a decrease in negative affect. Affinity has been shown to increase civic engagement. Thus, we conclude that affiliative smiles displayed by leading candidates during the campaign likely acted as a mechanism to align voter behaviour with the dominant political message.

## Introduction

A considerable body of experimental work has explored the perception of leadership judgements from various nonverbal cues that may convey emotions [1–3]. Facial displays are a rich source of perceived leadership traits [4]. Various expressive cues are relied on heuristically to detect leaders—and appropriate leader behavior; indeed, observers can quite rapidly process trait-related information form facial displays and make accurate inferences about competence and other important dimensions of leadership [5]. This ability does not require advanced learning and is observed in children who can accurately recognize successful political leaders by comparing pairs of photographs, even when they have no prior knowledge of the leaders’ political background [6]. Interestingly, the less tangible evidence observers have on the effectiveness of an individual’s leadership abilities, the more they tend to rely on facial cues to make leadership judgments [7].

Two facial cues significantly drive leadership judgments: lower pupil-to-brow distance and smiles [8]. Individuals with smaller pupil-to-brow distance who also smile are perceived as effective leaders [9]. The intensity of the smile, especially at a specific encounter, plays a crucial role in processing leadership traits. The smile interacts with these judgments, and dominance is associated with smiling faces displaying a lower pupil-to-brow distance [10]. Little is known about the influence of different smile types on observer responses, however.

This is surprising, since it is now well established that such cues are key to the development and maintenance of leader-follower hierarchies [11]. Indeed, much is known about the mechanisms by which the emotional expressivity of leaders can shift the attitudes of followers [12]. Scholarly attention examining the effects of leader smiles have shown that factors such as authenticity, appropriateness and context can drive follower impressions [13]. This is especially pertinent for understanding how a politician’s smile can interact with voter attitudes, as the context of political debate is often marked by acts of competition and cooperation by protagonists.

Research has examined the impact of facial appearance on social outcomes, such as attributions of competence influencing U.S. congressional and gubernatorial election outcomes, and dominance inferences affecting military rank attainment [14–16]. Yet little work has specifically investigated how facial displays shape voter emotion in electoral contexts. To address this deficit, we study the 2019 UK general election as fertile ground to observe leader-follower response. A few years prior, a national referendum held in 2016 prompted a years-long convulsion of UK politics and society, due to the vote to leave the European Union (i.e., ‘Brexit’). Part of this process was the call for a general election in 2019, the result of which delivered a huge majority for the Conservative and Unionist Party under Boris Johnson, with the mandate to seek resolution of the Brexit process. This result was facilitated by a significant realignment of traditionally Labour-voting areas towards the Conservatives, and the failure of other major parties to mount an effective challenge.

A substantial body of research has focused on changing values in the electorate, especially towards surging support for populist candidates and platforms, and the failures of traditional parties and candidates to recognise and respond to the fissures opening in society [17]. However, it is not yet clear whether these shifts are attributable to voters’ changing ideologies and nationalistic beliefs, or whether there is an impact of more basic emotional responses to individual party leaders [18]. Intrinsic to the appeal of individual leaders are expressive cues that promote bonding and communicate reassurance, thereby enhancing electoral appeal [19]. Studies from the Reagan era established that, at the level of emotional response, smiles facilitate affinity and have the capacity to rally undecided and even other party supporters to a politically effective communicators [1].

Much of the empirical work on televised leader displays has investigated the expressive variation in and communication efficacy of candidate display repertoires in the context of political debates [20,21]. While debates are convenient to analyse, only so much insight can be generated from these structured settings. Indeed, candidates and office holders are portrayed and expected to appear in diverse environments, depending on the demands of the moment. To a large extent, the reception and influence of display behaviour depends on the context in which it occurs: whether candidates and office holders are competing for political power [22], engaged in governance activities or rallying the nation in the face of adversity or comforting followers in the face of loss [23]. While different behavioural intentions are reliably communicated through facial expressions (e.g., anger conveys threat, a fearful look conveys evasion), smiles hold a unique position in the panoply of facial displays as they can convey both affiliation, as well as reward cues, i.e., displays that are positive reinforcing to an observer [24].

The influence of smiles may be accentuated or attenuated depending on the relationship between the individual signalling and the observer receiving the signal [25–27]. In politics, followers are more likely to have a stronger empathetic response to leaders who espouse an ideologically consistent outlook than to those they oppose [28]. Supporters of US President Barack Obama, for instance, perceived greater happiness encoded in his different smile types than critics [29]. Although partisan identification leads to stronger empathy and counter-empathy in response to leader display behaviour [30], a more direct measure of support involves voting and taking electoral outcomes into consideration.

In prior work, we assessed viewer reports of happiness, affinity, anger, and distress in response to the reward and affiliative smiles, as well as ambiguous facial expressions, of party leaders Boris Johnson, Jeremy Corbyn, and Jo Swinson in the week before the December 2019 UK general election [31]. However, this work did not take into the account the complex social effects of the election itself. The outcome of which would likely have a different impact for the supporters of each of the different political parties. The current study extends this work by considering voter responses both before and after the election, allowing perceptions of winning or losing (and not just leading or trailing in the polls) to enter the picture. Reward and affiliative smiles are discussed below.

While some research has considered emotional responses to leader displays during the competitive period prior to elections, few studies have considered change after the electoral period. One such study did examine the effects of candidate smiles on voter emotions during the 2012 US presidential election between Barack Obama and Mitt Romney, finding that Obama’s followers reported happiness in response to his reward smiles, but no change in response to Romney’s displays. In comparison, Romney’s followers reported anger and distress to Obama’s facial displays in the election’s aftermath but minimal change in response to Romney himself [30]. Thus, it could be argued that as voters moved through the campaign period, early to late, election day, and post, emotional shifts occurred in response to the winning candidate’s displays, not the loser’s.

Differences in response to the facial displays of political winners and losers may also be driven by ideological proximity. Thus, the emotional response to candidates by followers, whether positive (happiness and affinity) or negative (anger and distress), may reflect closer or looser ideological alignment. The impact of this alignment should surface in relation to salient issues. For instance, it should be possible to examine the impact of Boris Johnson’s facial displays and attitudes towards Brexit in assessing candidate preference. Given the level of polarisation in Brexit-related attitudes that existed during the 2019 election, the facial displays of the political leaders may have served to drive voter proximity to key political messages.

While facial displays of anger and their threatening social intent can be used to bond supporters and assert dominance over rivals, the major means by which leaders obtain consensus, build support, and attract followers is through facial displays of happiness/reassurance [32]. These affinity building expressions encompass the array of smiles that not only signal the absence of threat, but are emitted to signal willingness to cooperate or occur when endogenous feelings of enjoyment are elicited [33,34].

To classify political smiles, we adopt Rychlowska’s taxonomy [34]. First, it should be noted that not all smiles are equal. While the contraction of the main *zygomatic major* muscle that pulls the lip corners up and at an angle is ubiquitous in all, there are a number of distinct smile types encompassing a range of nuanced muscle movements. Here, we assess two of these smiles, reward and affiliative, that largely correlate with ethological research in the political sphere showing they have distinct behavioural expectations and outcomes [29].

Reward smiles, also referred to as genuine or felt smiles, and in the animal behavior (ethological) literature as open mouth, bared teeth displays, are associated with playfulness, joy, and enthusiasm [35]. Such smile displays have high signal power, meaning they are the most likely to engender emotional contagion in observers. Consistent with this, exposure to reward smiles generates higher levels of self-reported happiness than other smile types [34]. Recent work on the social function of reward smiles has also documented higher levels of trust in response to these displays [36] Such a behavioural outcome would clearly have utility within political discourse where trust in politicians predicts civic engagement [37].

Finally, affiliative smiles communicate approachability, acknowledgement, and appeasement, and are associated with an affinity towards the individual at whom the display is communicated. While affiliative smiles also utilise the *zygomatic major* muscle, they often involve controlling the display through muscles around the mouth (e.g., the *obucularis oris*) and have a unique onset timing that is marked by sudden formation and rapid dissipation [36]. Perception of this smile engenders prosocial impressions that are an important foundation to cooperative relationships [32].

The dichotomy of behavioural outcomes associated with reward and affiliative smiles can be summarised as approach behaviour versus social bonding and cooperation, respectively. Such a dichotomy has relevance for effective politics where the *raison d’etre* for all political campaigns is to convince the populace to act in a supportive manner, whether voting, volunteering or other behaviour. Despite the wealth of studies on affiliative and reward displays, the affective response of voters to these smile types has not yet been shown.

If a politician’s smile is effective in shifting the affective reaction of voters, then the facial displays of the election winner should drive a distinct pattern of response compared to the loser’s. Prior research indicates that the efficacy of the winner’s facial displays may drive more positive response for losing candidate supporters than displays from their own party leaders. Here we would expect to see supporters of the winning party showing an increase in positive affect when they are presented with either reward or affiliative smile displays from their party leader. By contrast, exposure to reward smiles should not increase self-reported positive affect for losing party supporters.

Previous work has shown that simple partisanship may drive an increase in positive affect when voters are presented with examples of various smile displays from favoured candidates after an election [30]. However, these findings have only been explored in the bipartisan context of US presidential politics, so it remains an open question as to whether the same dynamic will materialise when there are more than two parties competing. In addition to the above hypotheses the role of ambiguous or neutral facial displays in shifting the affective response of voters will be examined in an exploratory fashion.

To test these hypotheses, individuals who declared their political allegiance to one of the three major political parties in the UK’s 2019 general election were invited to participate in an online experiment. Individuals who were undecided about the election were excluded. Participants were shown video footage of three prominent political figures: Boris Johnson, who was then leader of the Conservative Party—a center-right political party; Jeremy Corbyn, then leader of the center-left Labour Party; and Jo Swinson, then leader of the socially liberal-leaning Liberal Democrat Party. Participants for the study were recruited directly from a Qualtrics participant panel. They were shown a series of videos of each of the three political leaders responding to audience questions at a speech (see below for further details). The study utilised a pre/post-test design. To isolate election effects, participants were shown the same video footage of Johnson, Corbyn, and Swinson both before and after the 2019 UK general election. The purpose was to compare shifts in perceptions of expressive leader displays holding all else but the timing constant, as well as to identify any changes in attitudes towards the political figures. By using the same participant pool and presenting the same stimuli at two different time points, the study was designed to provide insights into how political perceptions and attitudes change over time, particularly in the context of a highly charged political environment such as a general election.

## Methods

### Stimulus material

Nine short (7–10 second) video excerpts of Johnson, Corbyn and Swinson were collected from the public domain. We have described the imagery in detail in our previous report and would direct readers to that source for a detailed description of the material [31]. To maintain external validity, any textual background or media icons superimposed onto the video were not removed from the frame. Excerpt choice was based upon each of the party leaders being presented in a standardized head and shoulder frame in front of a neutral background. All excerpts depicted the political leader either listening to constituent or journalist questions (Johnson and Swinson) or nonverbally responding to an observable audience response (e.g., laughter, applause, cheering, booing, or chanting; Corbyn). This facilitated the capture of uninterrupted and naturalistic facial display behaviour by the party leaders.

To ensure the intended displays were used as stimuli, all material was examined before fielding by two expert Facial Action Coding System (FACs) observers [38], who identified the various action units at the level of muscle movements that were present in the reward and affiliative smile displays as well as the ambiguous or neutral displays emitted by each politician. Here, interrater agreement occurred significantly above chance for Joanna Swinson (affiliative smile *k* = 0.69, 95% CI 0.50, -0.24; reward smile *k* = 0.52, 95% CI 0.39, -0.23) Jeremy Corbyn (affiliative smile *k* = 0.60, 95% CI 0.64, -0.34; reward smile *k* = 0.50, 95% CI 0.07, -0.04) and Boris Johnson (affiliative smile *k* = 0.64, CI 0.86, -0.44; reward smile *k* = 0.48, 95% CI 0.73, -0.44). Interrater reliability for the ambiguous facial displays failed to reveal any statistical differences (see S1 Table).

To assess the differences in complexity within the various smile categories, and provide an element of confirmation, 26 raters (23 female and 3 male, *M* age = 20 yrs, range = 18–24 yrs) were asked to identify the emotion displayed in each video and then rate its complexity. Participants were instructed to select one response from a forced-choice set of six possible facial display labels reflecting basic emotion categories: happiness, anger, disgust, fear, happiness, surprise, and sadness.

They were also asked to indicate the complexity of the identified emotion on a scale of 1 (minimal) to 10 (maximum possible). Here ‘minimal’ was defined as being a simple display with very little complexity and easy to interpret and ‘maximum’ was defined as being highly complex requiring interpretation. For the rewarding smile displays, Johnson received judgments from 25 raters who identified the display as a smile and assigned a complexity rating of 47%, which was similar to Swinson’s reward display, where 25 individuals recognized the display as a smile with the same complexity rating. Corbyn’s reward display was identified as a smile by 23 raters, who rated its complexity at 21%. In general, affiliative displays received fewer smile judgments compared to the reward displays. Johnson (12 raters judged the smile to be 6% complex), Swinson (11 raters judged the smile to be 6% complex), and Corbyn (11 raters judged the display to be 5% complex). No neutral faces were perceived as containing any smile displays.

### Procedure

All video material was converted to MPEG-4 format with a 5-second countdown timer and embedded in an online experiment. Below each short video was a set of emotion sliders from 0–100% intensity with the question ‘How does the person in the video make you feel?’. Video footage was presented in two blocks. In the first block, ambiguous or neutral facial displays were shown to observers. In the second block, reward and affiliative smiles from each politician were shown. Each participant viewed all nine videos in semi random order with footage of ambiguous displays shown first and the order of the politician and smile type randomly presented. All participants were invited to complete the study over a 24-hour period one week before the UK General Election (starting 5 December 2019) and again one week after (19 December 2019). The full dataset of participants who responded the week prior to the election is reported elsewhere (see [31]). Here, only those individuals who responded both before and after the election are considered.

### Participants

Due to the devolved nature of national politics in the United Kingdom, only individuals residing in England and who indicated they intended to vote for one of three main political parties were invited to participate in this study. To ensure a national sample, the test cohort consisted of participants from each of the 124 post code zones in England. To achieve this, participants were referred via a Qualtrics survey invitation to a participant panel. All received modest financial compensation for their participation (£5). A total of 344 participants took part in both stages of this study, 219 male and 125 female. The average age of participants was 57 years (range = 25 to 89). In the second phase of the study, all participants indicated that they had voted in the 2019 General Election and also indicated their partisanship with 200 (58%) indicating support for the Conservative and Unionist Party, 74 (22%) supporting The Labour Party, and 70 (20%) supporting The Liberal Democrat Party. To ensure test compliance and comparability, cases with completion times less than 7 mins or greater than 30 mins at both sample periods were excluded from subsequent analysis (44 participants total were excluded). All participants provided written informed consent prior to the first trial and all procedures were approved by the university IRB (Ref 1586).

### Measures

Measures of participant emotional response was assessed through eight slider bars arrayed vertically under each of the individual excerpts. The strength of the emotion felt was indicated when participants moved the slider bar across the screen (from left to right), progressing from “Not at all”/0 to “Extremely”/100 with the default setting being “Not at all.” The eight felt emotion terms were combined into four 100-point variables, including “anger” (angry + disgusted), “distress” (fearful + anxious), “happiness” (happy + excited), and”affinity” (proud + interested). The resulting Cronbach’s α for the scales ranged from 0.78 to 0.97 [31].

## Results

### Effects of Boris Johnson’s smile on voter emotion

Analysis for sphericity indicated a significant effect, *χ*2(20) = 1362, *p* < 0.001 with ε = 0.42; therefore, the degrees of freedom underwent the Greenhouse-Geisser adjustment. As hypothesised, the analysis revealed a significant three-way interaction between the three facial displays, partisan group, and the time point of the sample (either before or after the election), *F*(2.69, 400.4) = 4.19, *p* = 0.008, η 2 = 0.03. This interaction, coupled with the significant between-subjects effect of partisan grouping, *F*(2,297) = 5.44, *p* = 0.005, η 2 = 0.04, was interrogated further with pairwise contrasts for each of the groups (Table 1).

**Table 1:** Affective responses shown by partisan group pre- and post- the 2019 UK General Election in response to reward and affiliation smiles as well as ambiguous expressions made by Conservative party leader Boris Johnson. Mean and standard error of that mean are shown. Significant differences are indicated with an asterisk.

		Partisan Groups					
		Conservative		Labour		Liberal Democrats	
		Pre	Post	Pre	Post	Pre	Post
**Ambiguous**	Happy	38.25(2.33)	47.07(2.39)*	11.56(3.02)	13.96(2.89)	9.08(1.93)	8.99(1.92)
	Affinity	43.19(2.36)	50.10(2.39)*	13.42(3.03)	18.64(2.99)	12.08(1.95)	13.19(2.23)
	Anger	10.22(1.15)	8.91(1.11)	49.41(4.79)	59.50(4.72)*	44.43(4.38)	44.37(4.43)
	Distress	16.45(1.48)	12.70(1.25)*	48.83(4.60)	57.16(4.53)*	43.89(4.36)	44.52(4.25)
**Reward**	Happy	36.85(2.35)	46.077(2.42)*	12.06(3.08)	16.22(3.38)	8.38(1.71)	9.90(1.99)
	Affinity	40.20(2.38)	47.86(2.44)*	14.02(3.00)	21.31(3.54)*	9.61(1.70)	12.08(2.45)
	Anger	8.65(1.14)	7.23(1.05)*	43.38(5.13)	54.27(5.02)*	37.55(4.45)	42.13(4.24)
	Distress	12.25(1.30)	9.77(1.19)	44.81(4.95)	52.81(4.76)*	36.01(4.20)	40.89(4.25)
**Affiliation**	Happy	24.41(1.42)	35.20(2.47)*	22.88(2.55)	31.35(4.45)	11.09(1.71)	27.42(4.43)*
	Affinity	40.24(2.36)	36.84(2.50)	13.73(3.13)	30.92(4.35)*	9.73(1.74)	28.04(4.23)*
	Anger	7.61(1.13)	21.53(2.28)*	44.98(5.05)	33.26(4.97)	37.42(4.28)	21.71(3.83)*
	Distress	12.03(1.44)	22.35(2.24)*	43.81(5.01)	34.73(4.98)	39.00(4.31)	23.11(3.85)*

When shown Johnson’s ambiguous facial displays, Conservative party supporters revealed a significant increase in happiness, *t*(179) = 4.96, *p* < 0.001, as well as affinity, *t*(179) = 4.01, *p* < 0.001, and a decrease in distress, *t*(179) = 2.60, *p* = 0.01, after the election. Perhaps unsurprisingly, Labour supporters reported a significant increase in negative emotion when shown Johnson’s ambiguous facial displays post-election: anger, *t*(57) = 2.61, *p* = 0.01, and distress, *t*(57) = 2.12, *p* = 0.04, compared to Liberal Democrat supporters who showed no shift in their emotional response after the election.

When shown examples of Johnson’s reward smile, Conservative party supporters indicated a significant increase in self-reported happiness, *t*(179) = 4.45, *p* < 0.001, and affinity *t*(179) = 3.99, *p* < 0.001, after the election. This was in contrast to felt anger, which significantly decreased, *t*(179) = 1.96, *p* < 0.05. Supporters of the Labour party experienced an inverse pattern of response, reporting a significant increase in anger, *t*(57) = 3.27, *p* = 0.002, distress *t*(73) = 2.84, *p* = 0.006, and an increase in affinity *t*(57) = 2.58, p < 0.01. Liberal Democrat supporters showed no shift in emotional response when presented with Johnson’s rewarding smile displays post-election.

Conservative party supporters reported a significant increase in happiness *t*(179) = 3.90, *p* < 0.001, anger *t*(179) = 5.91, *p* < 0.001, and distress *t*(179) = 4.25, *p* < 0.001, when shown affiliative smiles, while Labour party supporters reported a significant increase in affinity, *t*(57) = 3.57, *p* = 0.001. Liberal Democrat supporters also reported a significant increase in happiness, *t*(61) = 3.72, *p* < 0.001, and affinity, *t*(61) = 4.46, *p* < 0.001, as well as a significant reduction in anger, *t*(61) = 2.99, *p* = 0.004, and distress *t*(61) = 3.02, p = 0.004, when shown Johnson’s affiliative smiles in the post-election period.

### Effects of Jeremy Corbyn’s smile on voter emotion

Initial analysis for sphericity revealed a significant effect, *χ*2 (20) = 1059, *p* < 0.001 and ε = 0.43. Therefore, as above, the data underwent Greenhouse-Geisser adjustment. The analysis of emotional responses when voters were shown Corbyn’s facial displays failed to reveal a significant three-way interaction (*p* = 0.6) but the analysis did reveal a significant between subjects effect, *F*(2, 297) = 3.92, *p* = 0.02, η 2 = 0.03.

Labour party supporters reported a significant increase in negative emotion, anger *t*(57) = 4.88, *p* < 0.001, and distress, *t*(57) = 3.87, *p* < 0.001, and a decrease in positive emotion when shown Corbyn’s ambiguous facial displays, happiness, *t*(57) = 2.15, p = 0.03, and affinity, *t*(57) = 2.86, *p* = 0.006. Supporters of the Liberal Democrats reported a significant increase in anger after the election, *t*(61) = 2.95, *p* = 0.004. Conservative party supporters showed increases in happiness, *t*(179) = 4.60, *p* < 0.001, and affinity *t*(179) = 4.18, *p* < 0.001, coupled with a decrease in distress, *t*(179) = 2.60, *p* = 0.01 (Table 2).

**Table 2:** Affective responses shown by partisan group pre- and post- the 2019 UK General Election in response to reward and affiliation smiles as well as ambiguous expressions made by Labour party leader Jeremy Corbyn. Mean and standard error of that mean are shown. Significant differences are indicated with an asterisk.

		Partisan Groups					
		Conservative		Labour		Liberal Democrats	
		Pre	Post	Pre	Post	Pre	Post
**Ambiguous**	Happy	5.10(0.57)	13.29(1.64)*	27.58(3.52)	18.19(3.41)*	12.58(2.50)	9.23(2.25)
	Affinity	7.45(0.76)	15.08(1.64)*	33.35(27.42)	20.62(3.47)*	16.82(2.41)	12.34(2.84)
	Anger	48.59(2.46)	45.01(2.48)	17.46(3.31)	42.36(4.73)*	23.80(3.71)	39.62(4.78)*
	Distress	51.63(2.48)	43.03(2.53)*	23.01(3.38)	44.53(4.55)*	33.38(4.00)	40.20(4.61)
**Reward**	Happy	7.73(1.01)	9.06(1.29)	33.95(3.97)	26.25(3.72)*	13.65(2.28)	11.75(2.17)
	Affinity	7.68(0.85)	8.84(1.08)	35.67(3.95)	31.93(3.89)	16.74(2.73)	12.42(2.10)*
	Anger	41.41(2.72)	44.60(2.70)	14.23(3.04)	25.68(4.08)*	23.31(3.95)	34.14(4.20)*
	Distress	45.79(2.59)	45.56(2.69)	15.93(3.10)	24.21(3.81)*	27.83(4.09)	34.37(4.14)*
**Affiliation**	Happy	9.59(1.21)	14.85(1.56)*	38.24(4.22)	17.26(2.84)*	19.13(3.12)	10.28(1.93)*
	Affinity	9.84(1.14)	15.86(1.69)*	40.24(4.26)	20.67(3.79)*	19.91(3.14)	10.23(2.47)*
	Anger	43.21(2.69)	41.36(21.60)	11.76(2.73)	37.73(5.09)*	20.71(3.65)	35.28(4.77)*
	Distress	47.80(2.64)	40.80(2.71)*	16.87(3.32)	35.85(4.75)*	25.93(4.03)	37.54(4.57)*

Corbyn’s reward smile engendered a significant increase in anger, *t*(57) = 3.22, *p* = 0.002, and distress, *t*(57) = 2.20, *p* = 0.03, and a concomitant decrease in happiness when shown to his supporters, *t*(57) = 2.06, *p* = 0.04. Liberal Democrat supporters also reported a significant increase in anger *t*(61) = 3.21, *p* = 0.002 and distress *t*(61) = 1.98, *p* = 0.05 when shown these smiles, but reported a decrease in affinity *t*(61) = 2.06, *p* = 0.04. Conservative party supporters did not reveal a significant shift in any affective response when shown Corbyn’s reward smile displays.

Finally, Corbyn’s affiliative smile elicited a decrease in positive emotion for Labour party supporters (happiness, *t*(57) = 4.24, *p* < 0.001, and affinity, *t*(57) = 3.54, *p* < 0.001) and an opposing increase in negative emotions (anger, *t*(57) = 4.82, *p* < 0.001, and distress, *t*(57) = 3.61, *p* < 0.001). Liberal Democrat supporters reported an increase in anger, *t*(61) = 2.71, *p* = 0.009, and distress, *t*(61) = 2.08, *p* = 0.04, and decrease in positive affect, happiness, *t*(61) = 2.52, *p* < 0.01, and affinity, *t*(61) = 2.46, *p* = 0.02. Conservative party supporters reported an increase in happiness, *t*(179) = 2.64, *p* = 0.009, and affinity, *t*(179) = 3.01, *p* = 0.003, and a decrease in distress, *t*(179) = 1,96, p = 0.05, in response to Corbyn’s affiliative smiles post-election.

### Effects of Joanna Swinson’s smile on voter emotions

Again the analysis of multivariate sphericity revealed a significant effect, χ2 (20) = 829.73, *p* < 0.001, ε = 0.5 and therefore underwent Greenhouse-Giesser adjustment. However, neither the three-way interaction between facial display, partisan group, and phase of the election nor the between subjects’ test of partisan groups was significant (all *p* > 0.3).

Liberal Democrat voters responded with less happiness, *t*(61) = 2.53, *p* = 0.02, and greater levels of self-reported anger, t(61) = 1.98, *p* = 0.05. when shown examples of Swinson’s ambiguous facial displays Of special interest is the finding that even Labour Party supporters reported an increase in anger and distress when shown examples of Swinson’s ambiguous facial display (anger, *t*(57) = 3.62, *p* = 0.001, and distress, *t*(57) = 2.06, *p* = 0.04). Conservative supporters also reported a significant increase in anger, *t*(179) = 2.15, *p* = 0.03.

Swinson’s reward smile also elicited anger, *t*(61) = 3.01, *p* = 0.004, and distress, *t*(61) = 2.44, *p* = 0.02, coupled with a decrease in positive emotion among Liberal Democrat supporters (happiness, *t*(61) = 3.23, *p* < 0.002, and affinity, *t*(61) = 2.96, *p* = 0.004). Similarly, Labour party supporters also reported an increase in anger, *t*(57) = 3.05, *p* = 0.003, and distress, *t*(57) = 2.31, *p* = 0.02, when shown examples of Swinson’s reward smile post- election. Swinson’s reward smile failed to elicit any shift in affective response for Conservative party supporters (Table 3).

**Table 3:** Affective responses shown by partisan group pre- and post- the 2019 UK General Election in response to reward and affiliation smiles as well as ambiguous expressions made by The Liberal Democrat party leader Joanna Swinson. Mean and standard error of that mean are shown. Significant differences are indicated with an asterisk.

		Partisan Groups					
		Conservative		Labour		Liberal Democrats	
		Pre	Post	Pre	Post	Pre	Post
**Ambiguous**	Happy	11.66(1.11)	12.74(1.254)	20.80(3.23)	17.06(2.31)	27.49(3.13)	21.61(2.94)*
	Affinity	12.68(1.21)	10.71(1.07)	22.78(3.10)	16.92(2.17)	29.55(3.07)	25.67(2.90)
	Anger	29.11(2.19)	34.03(2.15)*	20.65(3.18)	33.84(3.72)*	8.45(1.70)	12.62(2.04)*
	Distress	28.18(2.03)	31.95(2.12)	23.71(3.14)	30.29(3.51)*	17.70(2.35)	18.78(2.76)
**Reward**	Happy	12.63(1.31)	12.06(1.44)	18.39(2.96)	21.66(3.44)	34.87(3.70)	25.95(3.23)*
	Affinity	11.78(1.20)	11.59(1.36)	20.56(3.15)	20.30(2.95)	35.63(3.77)	26.10(3.36)*
	Anger	27.23(2.51)	30.33(2.39)	19.38(3.64)	29.50(4.06)*	4.40(1.04)	10.99(2.30)*
	Distress	30.25(2.41)	32.15(2.40)	20.57(3.73)	27.38(4.00)*	7.45(1.46)	14.24(2.72)*
**Affiliation**	Happy	7.43(0.76)	12.61(1.47)*	14.56(2.60)	13.17(2.67)	22.08(3.24)	11.45(2.30)*
	Affinity	8.41(0.86)	14.69(1.49)*	14.58(2.30)	15.99(3.17)	24.89(3.43)	10.82(2.15)*
	Anger	28.89(2.43)	33.25(2.34)	18.92(3.32)	27.28(4.29)	6.95(1.56)	18.67(3.43)*
	Distress	34.33(2.38)	35.61(2.34)	25.50(3.84)	29.41(4.06)	14.58(2.24)	24.38(3.62)*

As was the case with Swinson’s reward smiles, her affiliative smiles engendered a decrease in reported happiness, *t*(61) = 2.88, *p* = 0.005, and affinity, *t*(61) = 3.70, *p* < 0.001, among Liberal Democrat supporters, and a higher level of anger, *t*(61) = 3.37, *p* = 0.001, and distress, *t*(61) = 2.94, *p* = 0.005. While no contrast reached statistical significance for Labour party supporters, increases in self-reported happiness, *t*(179) = 3.00, *p* = 0.003, and affinity, *t*(179) = 3.54, *p* = 0.001, were observed for Conservative party supporters.

## Discussion

This study investigated how different smiles exhibited by British party leaders during the historic 2019 UK General Election swayed the affective response of voters. This specific election saw one party receive a gain in constitutional power, the degree of which had never been achieved. As such, it presented an opportune moment to examine the role that this powerful nonverbal cue has on driving affective response—the emotional bond between followers and leaders.

To explore possible mechanisms behind such a significant electoral shift, we examined the extent to which different smile types displayed by politicians drive changes in affective shifts among voters. Two smile types connected with distinct behavioural intentions, reward and affiliation, were shown before and after the election. Findings revealed that observers were significantly affected emotionally by the type of smile displayed and which politician evoked it. This adds to the body of literature that has previously shown facial displays of emotion, even impoverished ‘smiley’ emojis can have a significant effect in political cognition [39].

This was observed with significant interactions for partisanship, display type, and time of observation (pre- or post-election) but only for the winner–Boris Johnson. Corbyn and Swinson failed to significantly shift the affective response of voters during the election, and thus their appeal remained fixed and proved no match for Johnson’s charm.

The pattern of responses revealed here also provides insight into the effects of specific facial displays when they are aligned to a clear policy statement. Johnson’s repeated use of the statement ‘Get Brexit Done’ was one aspect of his performative style [40]. This served to garner public support as the national attitude at the time was one of frustration borne out of the almost blanket media coverage for the Brexit process [41]. On the other hand, Corbyn, who was the opposition leader in the UK political arena, championed a position that was almost completely defined by an ambiguous approach to Brexit. Swinson’s mandate was one that would ensure that the UK would remain in Europe. However, Swinson lacked the performative acumen that Johnson possessed and thus failed to make a significant impact on the electorate.

At the time, the electorate was faced with two candidates who failed to make a positive impact with their supporter base [42]. Indeed, findings from this and other studies support the notion that negative affect, including such emotions as distress and anger, are experienced by partisan supporters of the losing side in the wake of a pivotal election [43]. This affective shift is accentuated by the strength of partisan divides and, in the case of the 2019 election, by media framing of the campaign narrative along the lines of getting Brexit done or not, an issue that clearly favoured the Conservatives and which gave Johnson a proactive role, arguing in the affirmative [44]. Thus, not only did electoral momentum side with the eventual winner, but Johnson’s ability to take a positive stance towards the biggest issue to face British voters in decades resonated emotionally and lined up consistently with his nonverbal communication.

The increase in anger and distress reported by Labour party supporters in response to Johnson’s reward smiles is consistent with findings from the psychological literature on losing [45]. Previous work has shown that the supporters of the election loser show a decrease in various measures of wellbeing and even a rise in mental health issues and unhealthy behaviours following the election loss [46,47]. Indeed, such an effect was revealed across 25 European countries and was also driven by a clear partisan affiliation [48]. Here we add to that body of literature and argue that subsequent work in this area should delve further into the emotional consequences of losing in majoritarian as well as winner-take-all electoral systems. If conducted from a nonverbal communication perspective, such should consider the nuanced distinctions that occur between two very similar and slightly distinct smile displays. As the results from the current study suggest, this partisan effect is only elicited by a specific nonverbal cue–the reward smile.

Another interesting outcome of this study, with historical resonance back to the Reagan era, is the finding that Labour party supporters (similar to ‘Reagan Democrats’) reported a slight increase in affinity when shown Johnson’s reward smile. As is noted above this smile display engenders positive responses such as trust in various scenarios such a financial investments etc [see also 32]. Here the effect that this specific display has can be extended to the political arena. However, the finding that supporters of Labour, and not the Liberal Democrats, experienced such affinity may suggest a blurring of ideological lines. Consistent with Conservative party supporters who reported positive feelings of happiness and affinity [49], Labour supporters were on some level emotionally taken by Johnson’s performative campaign style, which may have influenced election outcomes [50].

Given that reward smiles have been shown to generate trust in subsequent interaction [36], it is interesting to compare the effects of Johnson’s facial displays on other partisan groups. Here, the possibility that affinity could generate behavioural intention that is aligned to an ideology is absent. Indeed, even when shown the reward display of Corbyn and Swinson, their followers did not report an increase in affinity at all.

On looking at the reward smile results, a distinct pattern of partisan-driven results emerge. The significant increase in affinity by Conservative party followers in response to Johnson’s reward smile is indicative of an ingroup partisan effect driven by an alignment to political ideology [51]. The spike in emotion in response to reward smiles provides added specificity to the already known tendency of partisans to respond favourably to leader facial displays [52,53]. This differential effect on partisan emotions for reward smile displays suggests something unique about this smile type that generates affinity when exhibited to voters in an election campaign, but only when aligned to the dominant ideology. Further research on this point is warranted.

Although the context of this study revolves around British politics, implications of this work are general enough to extend into other electoral contexts. Firstly, the ambiguous or neutral facial displays that were produced by each of the politicians produced a distinct profile of responses among partisans. Namely, after the election, Conservative party supporters reported more positive affect in response to Johnson’s neutral and ambiguous displays. Conversely Labour party supporters reported an opposite pattern of effects for their candidate, i.e., increases in anger and distress and decreases in happiness and affinity, when shown Corbyn’s ambiguous display. Liberal Democrat supporters largely mirrored the pattern of responses revealed for Labour supporters with an increase in anger and decreases in happiness and affinity. The finding of a partisan effect extends previous work in this area that has examined the emotional response to different forms of candidate smiles in the 2016 US presidential election [30]. However, the multi-party structure of UK politics extends our knowledge to a more complex political arena.

The relative paucity of differences in affective responses when comparing reward smiles to ambiguous displays further supports the notion that subtle differences in the range of muscular contractions around the face do indeed convey distinct social messages [54]. Indeed, Johnson’s reward smile was effective in driving increases in positive affect coupled with decreases in anger within a single partisan group, i.e., Conservative party supporters. The increase in reported affinity for Labour voters shows that the facial displays of an election winner positively impacted the supporters of the losing party. Such findings have implications for understanding the process by which effective transitions of power can occur after the results of the election are known. and further work in this area should focus on the possible role of this smile type with a larger participant sample size.

However, when considering Johnson’s affiliative smile displays, the results did not show a partisan effect. Both Labour and Liberal Democrat supporters reported significant increases in positive affect on exposure to affiliative displays from their political opponent. This may seem surprising, but early ethological studies on affiliative smiles also showed that this type of facial display can engender real world benefits that are attributed to a form of reciprocal altruism [55]. Further work from studies of economic games show that presentation of the affiliative smile produces a greater desire to manifest cooperative behaviour [56]. Here the data reveal that observers of Johnson’s affiliative smile display were showing positive emotional responses despite existing across different partisan groups.

The first clear effect of the affiliative smiles are the significant increases in positive affect revealed in each of the three partisan groups when participants were shown Johnson’s affiliative smile. Indeed, when one considers the entire profile of results across all partisan groups, Labour and Liberal Democrat supporters reported *increases* in anger or distress when they were presented with smiles from their respective party leaders. Such was the effect of Johnson’s smile display that it also had the effect of producing a significant reduction in anger and distress in supporters of the Liberal Democrat party which was in direct contrast to the increase in negative affect and the decrease in positive affect revealed when this group was shown an affiliative smile for their respective party leader (see S1 Fig).

The second point about affiliative smile displays concerns the effect on Johnson’s supporters, who reported a significant increase in positive affect when shown affiliative displays from each of the other party leaders. This finding suggests a ‘victory effect,’ where the supporters of the winning party of an election reveal more satisfaction in comparison to other partisan groups [30].

An additional and intriguing speculation is a kind of dissonance effect that would have arisen with the that would have been predicated by both the national frustration felt towards politicians in general and Brexit specifically [57]. Johnson’s clear political manifesto to complete the Brexit process may have driven the increase positive affect that was revealed across the partisan groups. After years of indecision about whether and how to implement Brexit, there could have very well been a general desire for renewed certainty and sense of completion [41]. Indeed, it is difficult to interpret the results of this study outside the influence of the Brexit process.

The inclusion of participants with strong political allegiances a week before the election was a deliberate aspect of the experimental design. Future work in this area should examine the responses of a larger and more representative sample of participants, including politically independent voters. Here, the effect of smile displays and shifts in voting behaviour may be elucidated fully. Another refinement would be to compare the responses of pre- and post-election responses with participants who take part after the election only. The introduction of this additional control group would exclude the slight possibility of a potential learning effect occurring due to simple exposure to the survey at the first time point.

While we have isolated effects of a particular expressive display (smiles) in this study, in real world settings work alongside a broad repertoire of other display types common in political competition, such as anger and contempt. Future work should explore the role that a wider range of facial displays can have in driving observer emotions in this context as well as the longevity of any shifts in partisan beliefs.

Such work would then be able to identify a possible hierarchy of affective displays that can be used to better understand the psychology of group behaviour around elections.

The campaign arena in the build-up for an election is a period defined by a significant increase in media coverage of the various contenders [58]. While the current study provides evidence that specific smile types sway the affective response of voters, more work is needed to document how smiles work in conjunction with other displays to not only generate affinity in voters but also convey social dominance to leaders.

The 2019 General Election saw the rise of a systematic and central populist approach to politics in the UK, replicated in a number of other Western democracies in recent years. Given the highly charged nature of populist campaigning, it goes without saying that populist mobilisation is dependent on the affective response of the voting public [59]. However, such affective mobilisation only occurs when the emotional context is aligned to a specific ingroup ideology [60]. The current study adds to this body of literature insofar as we can now describe a differential pattern of affective responses that are engendered by unique smile types. In today’s contemporary political arena populism is a fundamental force, often associated with leaders who display stereotypical behaviours that tend to define their leadership style [61]. Johnson’s domination of the 2019 General Election gave him to a platform to demonstrate his own unique and, in this instance, effective style of nonverbal communication [62]. As the findings of this study show, an effective part of his communication repertoire was his use of facial displays to convey affinity, smiles in particular, which arguably made his supporters, but perhaps even opposition supporters, more content with the outcome post-election.

## Supporting information

S1 Fig Barcharts showing the mean affective response for each partisan group for both the reward and affiliative smiles of the three politicians across the 2019 UK general election. The top row represents the affective response made to the reward smile while the bottom row represents the responses made to the affiliative smile. Charts B and D represent the average negative response (anger and distress) while charts A and C represent the average positive response (happiness and affiliation). The partisan groups are indicated by the colour of the bars with voters supporters for Johnson being indicated by the blue bars, Corbyn supporters with the red bars and the individuals who indicated that support Swinson being represented by the yellow bars.
(JPG)

S1 Table Recognition profile for the various action units contained in the material used in the current study. The shaded blocks refer to the presence of a particular muscular contraction that was classified as specific action unit by two independent FACs raters (as indicated by columns 1 and 2). We are extremely grateful to Professor Jennifer Fugate of Kansas State University who agreed to act as the second rater here.
(XLSX)
