# Supplementary figures and images for: Winners and losers: Emotional shifts across elections are conveyed by a politician’s smile
Source: PLoS One. 2024 Apr 29;19(4):e0301113. doi: 10.1371/journal.pone.0301113 (PMC11057739; doi:10.1371/journal.pone.0301113)

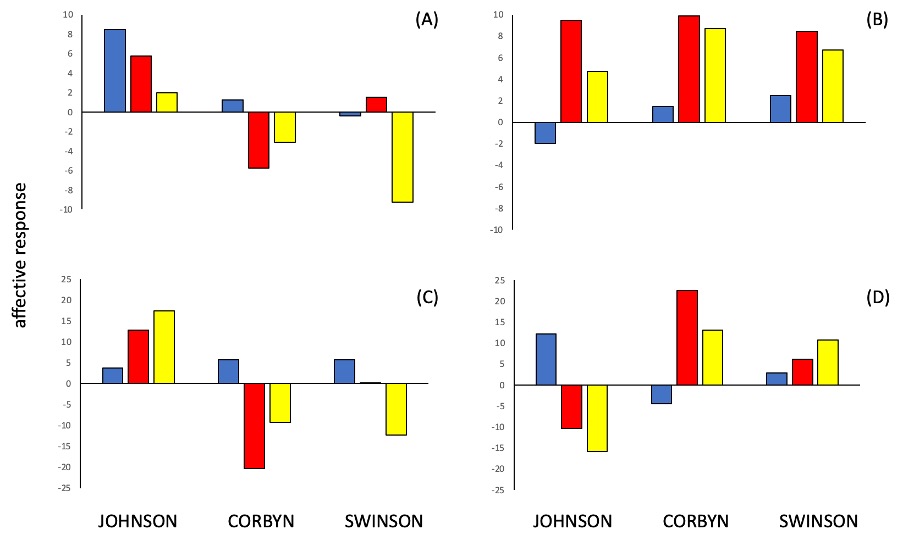

Supplement: S1 Fig — The top row represents the affective response made to the reward smile while the bottom row represents the responses made to the affiliative smile. Charts B and D represent the average negative response (anger and distress) while charts A and C represent the average positive response (happiness and affiliation). The partisan groups are indicated by the colour of the bars with voters supporters for Johnson being indicated by the blue bars, Corbyn supporters with the red bars and the individuals who indicated that support Swinson being represented by the yellow bars. (JPG) [file pone.0301113.s001.jpg]
